# Implementing paediatric early warning scores systems in the Netherlands: future implications

**DOI:** 10.1186/s12887-018-1099-6

**Published:** 2018-04-06

**Authors:** J. F. de Groot, N. Damen, E. de Loos, L. van de Steeg, L. Koopmans, P. Rosias, M. Bruijn, J. Goorhuis, C. Wagner

**Affiliations:** 1 NIVEL Netherlands Institute for Health Services Research, Otterstraat 118-124, 3513 CR Utrecht, the Netherlands; 2 Netherlands Federation of University Medical Centres-Consortium Quality of Care, NIAZ & CBOimpact Dutch Institute for Healthcare Improvement, Utrecht, the Netherlands; 3 Zuyderland Medical Centre Sittard, Sittard, the Netherlands; 4 Noord West Ziekenhuisgroep, Alkmaar, the Netherlands; 5 Medisch Spectrum Twente, P.O Box 50 000, 7500 KA Enschede, the Netherlands; 6 APH Amsterdam Public Health Institute, VU University Medical Centre, Amsterdam, the Netherlands; 7 Ecorys, P.O. Box 4175, 3006 AD Rotterdam, the Netherlands; 8 TNO Healthy Living, Schipholweg 77-89, 2316 ZL Leiden, the Netherlands; 9 Department of Pediatrics, Zuyderland Medical Center, PO Box 5500, 6130 MB Sittard, The Netherlands; 10 Department of Pediatrics, Northwest Clinics, P.O.Box 501, 1800 AM Alkmaar, The Netherlands

**Keywords:** Paediatric early warning score, PEWS, Implementation, Quality improvement

## Abstract

**Background:**

Paediatric Early Warning Scores (PEWS) are increasingly being used for early identification and management of clinical deterioration in paediatric patients. A PEWS system includes scores, cut-off points and appropriate early intervention. In 2011, The Dutch Ministry of Health advised hospitals to implement a PEWS system in order to improve patient safety in paediatric wards. The objective of this study was to examine the results of implementation of PEWS systems and to gain insight into the attitudes of professionals towards using a PEWS system in Dutch non-university hospitals.

**Methods:**

Quantitative data were gathered at start, midway and at the end of the implementation period through retrospective patient record review (*n* = 554). Semi-structured interviews with professionals (*n* = 8) were used to gain insight in the implementation process and experiences. The interviews were transcribed and analysed using an inductive approach.

**Results:**

Looking at PEWS systems of the five participating hospitals, different parameters and policies were found. While all hospitals included heart rate and respiratory rate, other variables differed among hospitals. At baseline, none of the hospitals used a PEWS system. After 1 year, PEWS were recorded in 69.2% of the patient records and elevated PEWS resulted in appropriate action in 49.1%. Three themes emerged from the interviews: 1) while the importance of using a PEWS system was acknowledged, professionals voiced some doubts about the effectiveness and validity of their PEWS system 2) registering PEWS required little extra effort and was facilitated by PEWS being integrated into the electronic patient record 3) Without a national PEWS system or guidelines, hospitals found it difficult to identify a suitable PEWS system for their setting. Existing systems were not always considered applicable in a non-university setting.

**Conclusions:**

After 1 year, hospitals showed improvements in the use of their PEWS system, although some were decidedly more successful than others. Doubts among staff about validity, effectiveness and communication with other hospitals during transfer to higher level care hospital might hinder sustainable implementation. For these purposes the development of a national PEWS system is recommended, consisting of a “core set” of PEWS, cut-off points and associated early intervention.

## Background

While paediatric resuscitation in hospitalized patients is rare, outcomes are poor [1]. Therefore, Paediatric Early Warning Scores (PEWS) are increasingly being used for early identification of clinical deterioration in paediatric patients. PEWS consist of a predefined set of parameters with age specific cut-off points. The idea behind PEWS is early recognition of clinical deterioration and thereby preventing mortality and/or morbidity related to resuscitation or avoidable deterioration of the child’s status. The calculation of PEWS is based on selected routine parameters in paediatric patients, such as heart rate, respiratory rate, respiratory distress, temperature, and oxygen saturation, but can also include information on behaviour and mental status [2–6]. A higher PEWS indicates a worse clinical condition and should ideally be followed up by early intervention by nursing and/or medical staff to prevent further deterioration [6].

Several authors, as well as the NHS Institute for Innovation and Improvement, argue that in order for PEWS to positively influence inpatient paediatric care, it is essential that they are part of a Pediatric Early Warning Scores S*ystem* [7, 8]. This requires, next to monitoring and registering symptoms and scores, clear guidelines for rapid decision-making on appropriate actions following elevated PEWS [6, 9]. Examples of such actions include calling a pediatrician or more frequent monitoring of the patient.

Because of the expected benefits of using a PEWS system and its role in optimizing safety and preventing avoidable damage in pediatric hospital care, the Dutch national patient safety program “Prevent Harm, Work Safely” in 2011 advised that all Dutch hospitals should implement a PEWS system. With this program, implementation of a PEWS system became part of the accreditation of Dutch hospitals (VMS Safety Program, 2011) [10]. At the same time though, the national patient safety program did not provide hospitals with guidelines regarding the content of the PEWS system to be used. E.g. which and how many parameters to use to calculate a score, which cut-off points to use, which patients to include, or a description of the associated actions as a result of elevated PEWS. Clear (inter)national standards for PEWS are lacking, which has resulted in multiple PEWS having been developed worldwide over the past decades. PEWS may differ regarding the included number of physiological and behavioural parameters and/or the cut-off points for intervention [5, 11].

During the same time of the VMS program in the Netherlands, a joint action was started within the European Union Network for Patient Safety and Quality of Care (PaSQ) (www.pasq.eu). This initiative aimed to support European hospitals in implementing several safe clinical practices, including the implementation of a PEWS system. In the Netherlands, five non-university hospitals participated in this PaSQ project from September of 2013 through December 2014 as an incentive to start the implementation of a PEWS system in their hospital.

The objectives of the current study were to evaluate the implementation of a PEWS system in five non-university hospitals within the European Union Network for PaSQ initiative in the Netherlands. For this purpose a mixed-methodology was used to 1) describe and compare the specific PEWS scores and protocols for early intervention (together a PEWS system) in each hospital; 2) evaluate the level of implementation of the PEWS system within each hospital, and 3) gain insight into the attitudes of nurses and paediatricians towards implementation of the PEWS system in their hospital.

## Methods

### Design and setting

Two of the participating hospitals were located in highly urbanized areas in the western part of the Netherlands, two were located in the south, and one was located in a more rural region in the eastern part of the country. See Table 1 for a description of hospital type and size.

**Table 1:** Description of included hospitals

	Type of Hospital	Number of beds	ICU present	PICU
1	General Hospital	425	Yes	No
2	General Hospital	633	Yes	No
3	General Hospital	290	Yes	No
4	Tertiary Teaching Hospital, non-university	1070	Yes	No
5	Tertiary Teaching Hospital, non-university	724	Yes	No

The goal of the five hospitals was to implement a PEWS system in at least one paediatric and/or maternity ward in their hospital. Due to a lack of a validated national PEWS system, hospitals were free to choose their own clinical symptoms to be included, scoring methods, cut-off points, and procedures following elevated PEWS. At the start of the PaSQ project, every participating hospital had developed a PEWS system, including a description of the selected parameters and associated actions for elevated PEWS, but none were actively working with a PEWS system.

The Dutch PaSQ team consisted of the Netherlands Institute for Health Services Research (NIVEL) and the Dutch Institute for Healthcare Improvement (CBO), with CBO providing the hospitals with implementation support throughout the project and NIVEL being responsible for the independent evaluation of the implementation. The PaSQ team organized national meetings for multidisciplinary hospital teams, as well as two webinars. The meetings were aimed at exchanging information on implementation progress, implementation training, and experiences in daily practice. CBO also held frequent individual conference calls with project leaders of the multidisciplinary hospital teams to discuss implementation progress and provide advice on how to carry out further implementation. The hospitals were made aware of the open accessible PaSQ website, the PaSQ implementation toolbox and PaSQ activities, such as webinars and exchange meetings. The online PaSQ toolbox consisted of information on the origin and background of PEWS, evidence on the effectiveness, and implementation advice. Additionally, a selection of specific tools, such as videos, checklists, and guidelines were provided, which could be used and/or adapted by health care organizations implementing a PEWS system. In order to provide participating hospitals with insight into their implementation progress, during the project NIVEL sent performance reports regarding the level of implementation to the hospitals at the start (September–October 2013), at midterm (May–June 2014), and at the end (November–December 2014) of the PaSQ project.

### Sample and data collection

In each hospital a paediatrician and paediatric nurse was appointed as contact person assisting the researchers in the organisation of data collection in the hospital. These contact persons also provided the researchers with the PEWS protocol as developed within their hospital, including chosen parameters, cut-off points, and local procedures. The independent evaluation of PEWS implementation consisted of two parts: 1) quantitative patient record review, and 2) qualitative semi-structured interviews.

#### Patient record review

A random sample of patient records was reviewed at the start, midterm and end of the PaSQ project. The sample consisted of records from discharged patients aged ≤18 years, with a length of hospital stay ≥24 h, admitted in October/November 2013, May/June 2014, or November/December 2014. We aimed to include at least 40 patient records per data collection period for each hospital, from either the paediatric or maternity/neonatal ward.

From these individual patient records, we determined the frequency of PEWS registration in the patient record, the number of elevated scores and the number of times that an elevated score lead to the appropriate action by nursing staff and/or medical staff as described in the hospital’s own protocol.

#### Semi-structured interviews

**Fig. 1:**
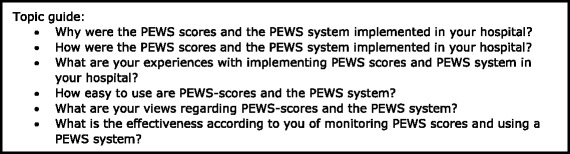
Topic guide for interviews with pediatric nurses and pediatricians

To gain insight into the implementation process and the attitude towards working with a PEWS system, semi-structured interviews were conducted during the second phase of the project (between January 2015 and March 2015) with the contact persons for the PASQ project. This meant that in each participating hospital one paediatric nurse and one paediatrician involved in the project was interviewed about the implementation process of PEWS scores and system at their paediatric ward. During these interviews, information was gathered on the method of introducing and implementing the PEWS system as well as their experiences with and attitudes towards a PEWS system (see Fig. 1 for topic guide). All interviews were held face-to-face and interviewees were assured that the gathered interview data remained confidential. With the participants’ consent, interviews were audiotaped for further analysis.

### Data analyses

Data from the patient record review were analysed using STATA 13.1. Descriptive statistics were used to describe hospital and patient characteristics, as well as the level of PEWS implementation per hospital.

Interview data were analysed using a grounded theory approach [12]. In doing so,, the interviews were transcribed verbatim into Microsoft Word. Next, open and thematic coding was used in order to identify generic themes related to the implementation of PEWS scores and the PEWS system. Two researchers (LK and LvdS) independently analysed the interviews, by coding the sections of the transcripts to find submerging themes. When coding disagreements arose, the researchers discussed the responses and selected the most appropriate code for each response. Qualitative data analyses were performed using MAXQDA 11.

## Results

### Description and comparison of PEWS scores and systems per hospital

Hospitals used different parameters and had different protocols for required action following elevated PEWS (see Tables 2 and 3). All hospitals assessed heartrate and breathing rate as part of the PEWS parameters, whereas four included respiratory effort (#1,2,4,5) or capillary refill time (#1,2,3,4) and three the use of supplementary O_2_. Other parameters, e.g. O_2_ saturation, temperature, systolic blood pressure, color, behavior and nurse’s concern were part of PEWS in two of the five hospitals. In existing literature, level of consciousness is often considered part of behavior, so combining these two items, behavior was part of the PEWS scoring system in four hospitals in our sample.

**Table 2:** List of items used in PEWS scores of the five participating hospitals

Item	Hospital 1	Hospital 2	Hospital 3	Hospital 4	Hospital 5
Heart rate	x	X	x	x	x
Breathing rate	x	X	x	x	x
Respiratory Effort	x	X		x	x
Capillary Refill Time	x	X	x	x	
Use of O_2_ therapy	x		x		x
O_2_ saturation	x		x		
Systolic Blood Pressure	x		x		
Temperature	x		x		
Colour		X		x	
Behaviour		X		x	
Consciousness			x		x
Nurse Concern			x		x
Total items	8	6	9	6	6

**Table 3:** Summary of PEWS systems used within each hospital

	General policy when to use PEWS	Cut-off points and associated actions
Hospital 1	High risk patient every 8 h PEWS ≤2 every 24 h	PEWS 4–5: assess PEWS every 4 h PEWS 6–7: assess PEWS every hour PEWS ≥8: consult pediatrician
Hospital 2	All patients every 8 h	PEWS 2: assess PEWS every 3 h PEWS 3: assess PEWS every hour and consult attending physician PEWS 4–5: call pediatrician and attending physician PEWS 6: consult with pediatrician within 30 min
Hospital 3	Patients on monitors every 8 h Other patients at least once, on admissions	PEWS ≥3: call pediatrician and assess PEWS every 2 h
Hospital 4	All patients every 8 h	PEWS 3: consider consulting pediatrician or attending physician PEWS ≥4: consult with pediatrician within 15 min
Hospital 5	All patients twice in 24 h	PEWS 3–4: re-assess PEWS after 4 h, consider assessment of blood pressure and O_2_ saturation PEWS 5–6: call pediatrician and measure blood pressure and O_2_ saturation

When looking at policies and required actions following elevated PEWS, most differences concerned the general policy for when to use PEWS, ranging from all patients every 8 h (#2 and 4), to all patients twice every 24 h (#5), to monitoring only on admission or patients on monitors and/or considered high risk (#1 and 3). In all hospitals, PEWS had to be calculated at least once for all paediatric inpatients. The cut-off points used by the hospitals all differed, but the actions of nursing staff following elevated PEWS would mostly include increasing the frequency of calculating the PEWS, consulting with a paediatrician and/or attending physician, or interventions aimed at directly improving the condition of the patient, such as administering oxygen or pain medication. With regards to the cut-off points, it is important to realize that PEWS scores are not comparable between hospitals as they are the results of different variables leading to a PEWS score.

#### Level of implementation in patients’ records

A total of 554 paediatric patient records were included in the study. One hospital (hospital # 2) dropped out during the last part of the implementation phase due to internal reorganization. A description of the patient characteristics can be found in Table 4.

**Table 4:** Patient characteristics of the reviewed records

Characteristic (*n* = 554)	
Sex (% male)	52.0
Mean age in years (SD)	4.5 (5.6)
Ward (%)	
Pediatrics	92.8
Maternity/neonatal care	7.2

Table 5 shows registration of PEWS at the different time points during the implementation. None of the hospitals were recording PEWS at the start of the study, and that at midterm only three hospitals (#2, #3 and #5) had started registering PEWS. One year after implementation, all hospitals had started to record PEWS. The registration of PEWS differed considerably, from 25.0% (#1) to 97.9% (#5), with an average of PEWS being recorded in 69.2% of the paediatric patients admitted (see Table 5).

**Table 5:** Registration of PEWS scores (n) at start, midterm and end of implementation

	Start (month = 0)	Midterm (month = 6)	End (month = 12)	Total records (n)
Hospital 1	0% (42)	0.0% (35)	25.0% (40)	117
Hospital 2	0% (42)	10.0% (40)	(0)	82
Hospital 3	0% (40)	72.4% (29)	61.4% (44)	113
Hospital 4	0% (45)	0.0% (33)	92.5% (40)	118
Hospital 5	0% (38)	92.3% (39)	97.9% (47)	124
Average	0% (207)	34.9% (176)	69.2% (171)	554

Next to PEWS being reported, elevated PEWS increasingly led to appropriate action as described in the hospitals’ own protocols (see Table 6). After 6 months of implementation, required action was taken in 10.3% of cases (3/29), whereas this number increased to 49.1% (27/55) after 1 year of implementation.

**Table 6:** Distribution of elevated PEWS and frequency of required action (PEWS system)

Month	Number of records	Registration of PEWS score	Action required^a^(n)	Action taken (n)
Start	207	0%	NA	NA
Midterm	176	34.9%	29	10.3% (3)
End	171	69.2%	55	49.1% (27)

^a^ = Based on information of the PEWS systems provided by the participating hospitals (see Table 3)

Overall, the patient records showed that after 1 year of implementation there was a positive trend towards improvement in using a PEWS system; PEWS were not only being recorded in more patients, but elevated PEWS led to appropriate action by the nursing and/or medical staff more often.

#### Interview findings: Implementation process and attitudes of professionals towards using a PEWS system

Eight semi-structured interviews were held with the contact persons for the project, consisting of paediatric nurses (*n* = 4) and paediatricians (*n* = 4) from the four remaining hospitals in the last phase. The mean age of the interviewees was 44 years (range 27–50 years) and on average they had 15 years of experience in paediatrics (range 6–27 years of experience). They spoke from both personal experience as well as experiences from colleagues within their department. The findings from the interviews will be presented here, grouped into three themes that emerged from the interviews regarding the attitudes of the healthcare professionals towards and experiences regarding the implementation of the PEWS system in their hospital.

### Theme 1: Using a PEWS system is important, but…

Most interviewed paediatric nurses and paediatricians were introduced to PEWS because it was part of the national VMS program in the Netherlands. They indicated that they already had a high registration burden, and showed some resistance to having to register yet another score. Nevertheless, they were motivated to use a PEWS system, indicating that they did acknowledge the importance of registering PEWS scores and working with a PEWS protocol for early intervention.

*“I think using the PEWS has added value. Just this week we had a boy who looked good clinically. But then we measured his blood pressure, and realised it was really low. He appeared to have hypotension. So yeah… we would not have noticed that otherwise, with only temperature and pulse. […] We always call each other a lot. Our paediatrician is easily approachable.”*

Nurse and care coordinator

Despite the fact that they considered working with PEWS important, they also voiced doubts about the effectiveness of using a PEWS system. Interviewees indicated that they considered evaluating the effectiveness of using the PEWS system as the next priority. They wondered how many children had been identified only because of an elevated PEWS, that would otherwise not have been identified.

*“I think that when a child is unstable, the doctor is often already alerted. […] When you are worried or it does not feel right, you warn the doctor. That does not rely on a score. It is not that I only start calling the doctor when I have a PEWS of 3-4-5, because you probably already did so because you are worried about the child. So in that respect, I do not think that we call the doctor faster by using PEWS-scores.”*

Nurse

*“I am not sure whether using PEWS really has an added value. […] We now want to investigate whether we are really able to identify those children at risk. Are there a lot of false positives? What could be the disadvantages of using PEWS? I do not immediately have a positive feeling about it. I do know that I almost never get called with the request ‘the PEWS is elevated, will you come by?’”*

Pediatrician

Although interviewees indicate that a PEWS can help in early identification of clinical deterioration in paediatric patients, early identification should not depend solely on elevated PEWS. Easily approachable nurses and physicians, as well as good communication, were considered to be vital for timely intervention in cases of clinical deterioration in paediatric patients.

### Theme 2: Easy to register and calculate especially when using an integrated system

In general, interviewees found the PEWS were easy to calculate because the underlying measurements were often already part of nurses’ daily work routine, and therefore required little behavioral changes in their daily work. Some interviewees found that exclusion of the peripheral blood pressure measurement would made the PEWS easier to use. Some quotes to illustrate these findings:

*“….. So I think that some people, at least that is what I heard in the beginning, were like ‘yet another score’. Of course you look at the child and you pay attention to the child, but you spend so much time behind the computer just to register everything. That creates resistance sometimes. I hear that, and I think: ‘yes of course you can see when a child deteriorates, but the registration, and being able to find it back in the computer, is important’.”*

Nurse

*“[…] I am under the impression that measuring PEWS does not require a lot of extra work from the nurses.”*

Pediatrician

Once the PEWS scores and the protocol for early intervention were selected, the implementation of the PEWS system showed few difficulties and improved when nurses were aware of the reason why PEWS scores were calculated and realised that it was not a lot of extra work.

Facilitators for the implementation of registration of PEWS included the integration of PEWS scores into the electronic patient records. While this effort was time consuming and difficult, it proofed worthwhile to facilitate implementation.

*“It took a very long time to embed PEWS in our department. Why? On the one hand I think that we did not clearly formulate the purpose of measuring PEWS to the team. The intention we had concerning PEWS was unclear and everyone thought it made the workload heavier: another extra activity. […] But now, in hindsight, you realise that you already do perform many of these measurements on a daily basis. But now you put them in a format, which you didn’t do before. So, actually it only takes you a very short time, but instantly gives you an idea of the child’s situation. […]”*

Nurse

*“We also connected the acute medication list to filling out the PEWS. So, when we enter the child’s information, it then provides a list that assists the nurses. This way nurses are motivated to first obtain and enter the score and in order to get that list. It provides information and lets you know how much and what you can give. Almost everyone does that. But whether it is really done a couple of times a day, I cannot say.”*

Care coordinator

One hospital (#1) had not included PEWS in their electronic patient records yet, but interviewees from this hospital indicated that this was desirable.

### Theme 3:National guidelines versus local applicability

The implementation advice and practical tools provided during the first face-to-face meeting were taken to heart by several project leaders and applied in practice. This led to project leaders involving the end users in selecting specific PEWS variables and developing a project plan for their hospital.

Hospitals found it difficult to identify a suitable PEWS system for their setting. The nursing and medical staff felt that the available systems were all developed for use in university hospitals, making them not necessarily applicable in their non-academic setting. This was partly due to the absence of an paediatric intensive care, where for instance intra-arterial measurements and monitoring are often used to calculate PEWS. In addition, nurses and paediatricians indicated that they would have liked to be provided with one national, uniform PEWS system that all hospitals could use. Because such a national system as lacking, all hospitals searched for their own suitable PEWS system. Most hospitals did so on their own, for example by instating a working group, by researching the literature, or by asking information from other hospitals (including regional university medical centres).

*“At first we decided to await a national development of a PEWS system, so we didn’t have to “re-invent the wheel”. But that took a very long time and we had to get started with PEWS in our hospital, so we decided to set up our own PEWS. […] Of course every nurse wants to identify that one child you would otherwise miss. But having to fill in something, again, like with the pain score… All the checks you have to do create resistance.”*

Nurse

## Discussion

The objectives of the current mixed methods study were to 1) describe and compare the specific PEWS and protocols for early intervention (together a PEWS system) in each hospital; 2) evaluate the level of implementation of a PEWS system within each hospital and 3) gain insight into the attitudes of nurses and paediatricians towards implementation of a PEWS system in their hospital.

While implementation of a PEWS system became part of accreditation of Dutch hospitals, national guidelines regarding which clinical symptoms to be included, which scoring methods, cut-off points, and procedures to use were lacking. Therefore, it came as no surprise that the five hospitals all developed their own criteria, scoring and policies regarding the use of PEWS. A recent study showed that in 68 Dutch hospitals that had implemented a PEWS system, 45 different versions were being used, including 200 different parameters, with not one parameter being used in all hospitals [13]. Using different parameters, elevated scores are not necessarily comparable between hospitals. This situation, of each hospital developing or adapting their own PEWS criteria and policies, is similar to the situation in the United Kingdom and North America [6]. On one hand, different contexts and clinical populations justify different PEWS variables, but at the same time, these differences are barriers to communication between hospitals and the developing evidence for effective and validated systems [8]. This was also reflected in the doubts by the interviewed professionals, while they intuitively thought of PEWS system as a positive value in the daily care of children, they also voiced doubts about the validity and effectiveness of registering PEWS scores. This clearly indicates the need for nationally agreed guidelines when developing a PEWS system.

While PEWS have been validated for more specific settings such as university hospitals [9], questions can be raised regarding the use of the same list of parameters in other settings, such as non-university hospitals. At the same time however, most of our included hospitals did base their parameters on internationally published PEWS, such as the “Brighton PEWS-score” [3, 14], using parameters concerning the cardiovascular and respiratory system and behaviour. While for the point of view of validating and evaluating effectiveness, one standard set of PEWS parameters might be preferred, it is important to keep in mind that non-university settings often include different populations of patients and thus a different a-priori chance of developing acute deterioration based on physiological markers. This was also reflected in the interviews with professionals. While they acknowledged the importance and the purpose of PEWS, they also expressed doubts regarding the effectiveness of the PEWS in their own hospital. They felt the parameters of PEWS were developed in and for university or specialized hospitals with more complex pathology. Because acute clinical deterioration was a rare occurrence in the participating hospitals, the nurses and paediatricians felt that they had not yet the opportunity to observe and experience the usefulness of PEWS in practice.

For example, elevated temperature and heart rate in an otherwise healthy child could carry a very different risk of further escalation than those same symptoms in a child after bone marrow transplantation at a specialized oncology unit. The solution for this tension between on one hand PEWS variables that are appropriate for specific settings and on the other the call for a more standardized PEWS, could be to develop a core set of PEWS that can be used effectively in all settings, while still leaving room for specific populations and different settings. The development of a core set of PEWS and standardized protocols for follow up to be used in all Dutch hospitals would also enable these hospitals to more easily compare results and exchange information on quality improvement efforts. In the Netherlands, the Dutch Society for Paediatricians (NVK) and the Dutch society for Nurses (V&VN) are collaborating in a national working group to further support the development of a core data set for PEWS scores and early intervention for different settings.

Looking at the success of implementation, a couple of things stand out. Findings from the patient records seem to indicate that the registration of PEWS had improved during the PaSQ project, although the extent of implementation differed across hospitals ranged from 25 to 98%. While the staff was motivated to implement a PEWS system, because they believed in the goal of the PEWS system, they had doubts about whether the currently used often non-validated scores and protocols would lead to the desired outcomes. In contrast to studies from the United States reporting that the introduction of PEWS improved nurses’ and paediatricians’ abilities to recognize early signs of deterioration [15, 16], the interviewed professionals in our study were not convinced of this effect. Nurses and paediatricians indicated that prior to the introduction of PEWS, they already communicated effectively regarding the deterioration of patients, without experiencing hierarchical boundaries. This may point to a cultural difference between countries in how nurses and pediatricians work together.

These doubts and lack of obvious better ability to detect clinical deterioration could be part of the reasons why still more than 50% of the elevated scores did not lead to action as required by the local hospital policy. Therefore these doubts from professionals should be taken seriously in future improvement projects, as attitude and believes are important factors in implementation and sustainability of using a PEWS systems in non-university hospitals [17]. These concerns are supported by the mixed existing evidence regarding the effectiveness of using a PEWS system, with some studies showing positive effectiveness of PEWS in improving patient care, while other studies showing no positive effects [18, 19].

On a positive note, interviewees indicated that the relative ease with which a PEWS system could be incorporated in daily practice, facilitated implementation. Because many of the measurements underlying PEWS scores were already part of daily routine - and thus familiar to nurses - the practical use and registration of PEWS required little extra nursing effort, while the administrative burden of entering them into a system was perceived as high in some hospitals. A good digital system for entering and analysing the PEWS was mentioned during the interviews as a facilitating factor for implementation. This was in line with our findings from the patient record review. The two hospitals showing almost 100% implementation of PEWS (hospitals 4 and 5) were those where PEWS were incorporated in the electronic patient records. At the same time, these two hospitals were the two tertiary teaching hospitals in the sample, often dedicated to more complex care. In the Netherlands, general hospitals provide good quality basic care, where the non-university tertiary teaching hospitals and university hospitals provide more complex and specialized care. While all hospitals in the sample had access to an ICU, only the eight university hospitals have a paediatric ICU. This means that when a patient deteriorates or when PEWS are elevated, the child may have to be transferred to a higher level care hospital. During transition of care, good communication is essential for continuation of care [20]. With hospitals all using their own set of parameters and scoring procedures, important information may be missed during an acute transfer. This is another reason, professionals argue for the development of a core set of PEWS scores for Dutch hospitals.

Recent literature on change management research emphasizes the importance of small changes to existing behaviours in determining the success of implementation of new guidelines and protocols. The smaller the differences between current work processes and the new work processes [21], the easier the implementation process. In our study, successful implementation required very little change from nurses, whereas the less successful hospitals required nurses to use different systems for the administration of PEWS and the administration of regular patient information. The results of this study also shows less complex behaviour, in this case recording the PEWS, was easier to implement than the more complex changes, e.g. implementation of the early intervention protocols following an elevated PEWS score.

What we also learn from this study is that implementation of new policies takes time. At 6 months PEWS were registered in 34.9% and elevated PEWS led to appropriate action in 10.3%, whereas at 6 months this number increased to respectively 69.2% and 49.1%.

This study has several limitations. One of them being the methodology of using patient records. While this method is being used extensively in paediatric patient safety research [18, 22, 23] and has been proven a reliable way to assess patient safety [24], it is limited by the information that is actually being recorded by the healthcare professionals and by the quality of the patient records. Therefore, it is recommended to include different sources of information when evaluating (paediatric) patient safely issues [25]. For that reason we did enhance the patient record data with interviews with representatives of the wards and used the individual hospital protocols in the analysis. Even using a mixed methods approach, it is still difficult to understand the less than 50% rate of follow up after elevated PEWS. Recommendations for future research into the effectiveness of PEWS include a more process oriented evaluation, including experiences by patients and parents, patient safety culture assessment throughout the wards and using cases from where PEWS might have “saved” a child instead of the focus on adverse events which in paediatrics are small in number [18].

Secondly, the outcomes of the study are limited to a small sample of non-university hospitals, limiting generalizability of these results. At the same though, the number of patients included in this study was high (*n* = 554) and the hospitals were selected from different regions and different levels of urbanization and therefore a representative mix of non-university hospitals in the Netherlands.

## Conclusion

Hospitals participating in the PaSQ project showed improvements in the use of their PEWS system, although some were decidedly more successful than others. Facilitating factors included the small amount of change in professional behavior that was required, in combination with a system integrated into the EPD for entering and analysing the PEWS. However, doubts among staff about validity and effectiveness might hinder further sustainable implementation. More evidence is needed regarding effectiveness of using a PEWS system. For this purpose the development of a national PEWS system is highly recommended, existing of a “core set” of PEWS and clinical decision making rules for elevated scores in different clinical settings.

## Abbreviations

CBO: Dutch Institute for Healthcare Improvement
NIVEL: Netherlands Institute for Health Services Research
NVK: Dutch society for pediatricians
PaSQ: Patient Safety and Quality of Care
PEWS: Paediatric early warning scores
V&VN: Dutch Society for Nurses
